# Listening to Slugs: Acceptability and Consumption of Molluscicide Pellets by the Grey Field Slug, *Deroceras reticulatum*

**DOI:** 10.3390/insects12060548

**Published:** 2021-06-11

**Authors:** Samantha Mirhaya de Silva, David Chesmore, Jack Smith, Gordon Port

**Affiliations:** 1 School of Natural and Environmental Sciences, Newcastle University, Newcastle upon Tyne NE1 7RU, UK; Gordon.Port@newcastle.ac.uk; 2 Department of Electronic Engineering, The University of York, Heslington, York YO10 5DD, UK; dchesmore@googlemail.com (D.C.); jack.a.smith@live.com (J.S.)

**Keywords:** Grey Field Slug, *Deroceras reticulatum*, invertebrate bioacoustics, acceptability, consumption

## Abstract

**Simple Summary:**

The Grey Field Slug, *Deroceras reticulatum* (Müller), is a common crop pest worldwide. To control slugs, chemicals, such as metaldehyde are incorporated into pellets which are toxic to slugs when consumed. Our aim was to compare slug feeding on new pellet types with those already commercially available. Novel pellet types included silica-coated commercial metaldehyde pellets where metaldehyde is released more slowly. An audio sensor was used to record the individual slugs feeding on a variety of pellet types, including toxic (metaldehyde and ferric phosphate) and non-toxic (cereal) pellets. Two types of experiment were conducted: shorter laboratory trials and longer arena trials. The length of each bite and the total number of bites were recorded. There was no difference in the length of the bites between pellet types in laboratory trials. Novel pellets were not consumed more than commercial pellet types. Commercial pellet types did not differ in consumption.

**Abstract:**

Gastropod damage to crop plants has a significant economic impact on agricultural and horticultural industries worldwide, with the Grey Field Slug (*Deroceras reticulatum* (Müller)) considered the main mollusc pest in the United Kingdom and in many other temperate areas. The prevailing form of crop protection is pellets containing the active ingredient, metaldehyde. Metaldehyde can cause paralysis and death in the mollusc, depending on the amount ingested. The paralysing effects may result in reduced pellet consumption. A greater understanding of metaldehyde consumption may reveal an area that can be manipulated using novel molluscicide formulations. Novel pellet types included commercial metaldehyde pellets coated so that metaldehyde is released more slowly. In both laboratory and arena trials, an audio sensor was used to record individual slugs feeding on a variety of pellet types, including commercially available toxic pellets (metaldehyde and ferric phosphate) and novel metaldehyde formulations. The sensor was used to record the length of each bite and the total number of bites. There was no significant difference in the length of bites between pellet types in laboratory trials. Novel pellets were not consumed more than commercial pellet types. Commercial pellet types did not differ in consumption.

## 1. Introduction

Agricultural and horticultural industries are keen to manage pest slug species, mostly to prevent yield loss and due to marketing standards for produce to be “practically free from pests… [and] from damage caused by pests” [1]. Growers usually employ a variety of methods to limit slug damage to crops, the most prevalent being the application of metaldehyde pellets [2]. Metaldehyde pellets kill pest molluscs or paralyse them, making them more vulnerable to predators and environmental stresses [3]. Poor application or rainfall events can cause metaldehyde to leach from the pellets into the water system where it is difficult and expensive to remove [4,5,6]. Metaldehyde has consistently been the main cause for failure to achieve European Union drinking water standards of 0.1 μg L^−1^ for individual pesticides and 0.5 μg L^−1^ for total pesticides [7,8]. By March 2022, metaldehyde slug pellets will be withdrawn from widespread use in the UK, but they are still in use globally [9]. 

Slug pellets are made of a cereal base to encourage slug feeding, dye to discourage the ingestion of the pellet by non-target species and compounds (stabilisers, binders, fungicides) to extend the longevity of the pellet. Some pellets may include compounds that aim to attract slugs towards the pellet or prolong feeding on the pellet [10]. Weather conditions and distribution onto the soil, amongst other factors, have a cumulative effect on the effectiveness of the pellet [11,12]. Pellets can cause paralysis or death depending on the amount consumed; there also may be minor adverse reactions if contact only is made with the pellet [13,14,15]. Poor consumption together with favourable environmental conditions may allow slugs to completely recover [16]. Mortality is reliant upon consumption by the slug. Greater understanding of the slug–pellet interactions are essential to improve pellet efficiency.

An “attractant” is a substance that would encourage the slug, from a distance, to move towards the food source, more so than a food source that did not contain that attractant [17]. While slugs forage randomly, at shorter distances, they may be able to detect food [18]. There is insufficient work to define a distance at which slugs may be able to detect food sources and this may vary between species [19,20,21]. An attractive food source may cause the slug to move towards it, while “repellent” properties may cause the slug to move away [17]. Pellets may contain a substance that is attractive (cereal) as well as compounds that may repel the slug. 

Upon physical contact with a potential food source, slugs may take a few test bites to determine the “acceptability” of the food—the likelihood of the slug feeding after initial physical contact with the pellet. If the pellet is accepted, the slug may continue feeding depending on the ‘palatability’ which refers to the effect of ingredients on consumption [17]. Certain substances can increase or decrease the feeding response at each stage of feeding, as well as impact digestion [22,23,24,25].

There is much literature available comparing the effect of different food types on slugs using various methodologies, but measuring the amount of food consumed is difficult [16,26,27,28,29]. Water evaporation and mucus contamination means there is no reliable way to measure the weight of the pellet in an open system where the slug is free roaming. It is also not feasible to weigh the slug due to the reliance of slug weight on evaporation [30,31]. When a slug feeds, food fragments are broken off by the buccal mass, the organ responsible for coordinated feeding and mastication [32]. Sounds of the food fragments being broken off can be recorded and quantified. Due to the difficulties in measuring weight, acoustic information produced during consumption can be used as a proxy, which allows the comparison of different pellet types. The amount of substance a slug has consumed is important information for pellet developers and manufacturers, particularly to establish whether feeding occurs at all. 

Acoustic techniques to quantify bite data were first used by Wedgwood and Bailey (1986) [33]. Pellets were glued to a piezo-electric gramophone pick-up, the disturbances were viewed on an oscilloscope, and the recording was played back on an audiocassette. A very similar method for recording the audio was used for later experiments by the same authors, but instead of an oscilloscope and audiocassette, a BBC microcomputer was used [34,35,36]. Audio and video technology have improved considerably since the publication of this initial bioacoustics work. The methodology developed in this paper produces more detailed data on slug consumption and was based on previous methods described above. More detailed data on slug consumption, acquired with an improved methodology, are presented in this paper.

Novel metaldehyde formulations use a silica matrix to coat pellets and therefore reduce the release of metaldehyde into water courses during rain events. The aim was to control the release of metaldehyde and to prevent slugs detecting the metaldehyde initially, and hence succumb to premature paralysis. In theory, this would allow more slugs to consume a lethal amount, resulting in a higher death rate and consequently less damage to crops. The overall objective of this paper was to assess the acceptability and consumption of experimental and commercial pellets, as a precursor for the successful commercial development of the novel products. Additionally, the methodology described in this paper may be used to provide further insight to any novel slug or snail pellet produced. Shorter laboratory trials aimed to provide a rapid and efficient method to compare acceptability and consumption between formulations. A longer arena trial aimed to investigate whether the environment in laboratory trials had an impact on the results, as well as explore if it would be possible to use this methodology under field conditions.

## 2. Materials and Methods

Two types of experiment were done. Laboratory trials involved recording the feeding of individual slugs to assess their feeding on all of the formulations, as shown in Table 1. Novel formulations were developed by Lucideon. Arena trials involved groups of slugs feeding on commercial formulations in a situation more similar to that where slugs encounter a pellet in nature.

### 2.1. Slug Collection and Maintenance

Slugs were collected using hand collection and chicken feed baited traps, from a variety of field sites around Newcastle upon Tyne (UK), approximately 36 h prior to the trials. Slugs were stored in plastic containers, lined with damp tissue paper to maintain humidity level, in a refrigerator (3–5 °C). The time spent in storage varied from 0 to 48 h, within and between experiments. Twenty-four hours before the experiment, the slugs were transferred to a controlled temperature room at 15 °C with a day: night cycle of 12:12 for 24 h. Pellets that were to be used in feeding were allowed to take up moisture from a damp filter paper under the same conditions (Table 1). Slugs were randomly assigned to a formulation for both laboratory and arena experiments. Slugs were removed from the controlled temperature room approximately 30 min before entering the trial. No slugs in the results described were preconditioned (no dummy pellets were used).

### 2.2. Laboratory Trials

**Figure 1:**
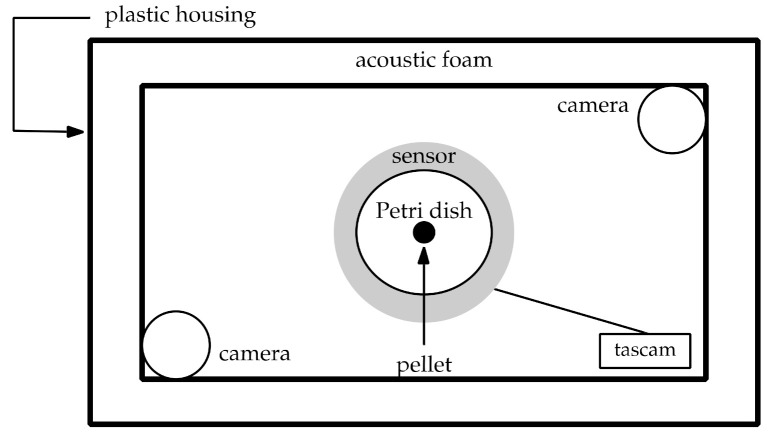
Audio and video equipment used to record bioacoustics laboratory trial experiments, not to scale.

A plastic box (57 × 39 × 28 cm) was used to house the recording equipment (Figure 1). Acoustic foam (10 cm acoustic foam panel—Advanced Acoustics AS4) lined the interior of the plastic housing and accompanying lid forming a sound-proof chamber. An audio-sensitive plate, from now referred to as the sensor, was used to record sounds produced. The sensor had an independent built-in power supply (PP3 9V battery) and was connected to a recording device (Tascam DR-05, TEAC Corporation, Guildford, UK). The sensor, power supply unit and Tascam DR-05 were placed on the bottom surface of the acoustic foam chamber. The apparatus was housed in an isolated room and no other work was conducted in the laboratory during the recordings. Simultaneous video recordings were taken using cameras (SCB_2001 video camera, Samsung, Chertsey, UK), an Inspire four-channel digital security recorder (INS-DVR04V2-250, Inspire, Oldham, UK) and an infrared light.

After the recording equipment was started, the Petri dish (9 cm) containing the pellet, glued to the base, was placed in the centre of the sensor and if necessary, the cameras were refocused on the pellet. Pellets were glued to ensure good contact with the base of the Petri dish and minimize noise. A single slug was placed in the Petri dish, and the locations of placement were constant throughout all trials. Then, the lid of the Petri dish was placed, upside down on the base, to prevent slug escape without hindering visibility. Each trial was allowed to run for 30 min regardless of pellet discovery or consumption. Laboratory trial recordings were analysed for ten minutes, which began immediately after the first bite—recordings where no bites were taken were not analysed any further. No slug fed on more than one pellet type. A new pellet and Petri dish were used in each trial. The identifying slug number, pellet type and experiment number were recorded before the start of each trial. After every third trial, a five-minute recording of the background noise level was taken, without a slug present (control recording). In order to maintain a stable temperature (17–22 °C) within the setup, the lid of the box was left open for at least 30 min before the next trial. Thirty trials were run for each formulation (Table 1) in a randomised block design. If any disturbance was noted, the trial ended, and a replacement trial was run afterwards. There were no observed mortalities.

### 2.3. Arena Trial

**Figure 2:**
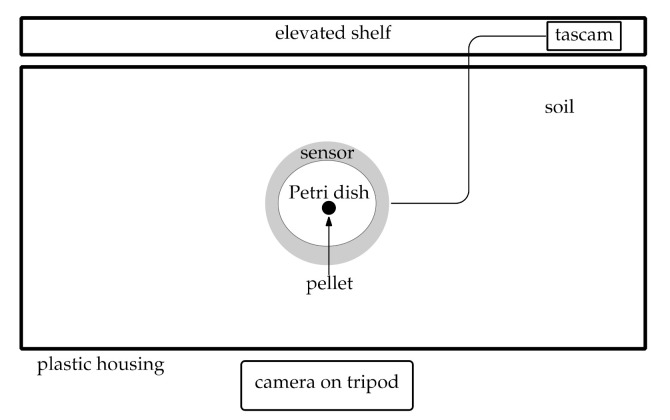
Audio and video equipment setup used to record bioacoustics in arena experiments, not to scale.

A plastic box (50 × 77 × 19 cm) was filled with soil (13 cm), which was collected from Cockle Park Farm, Morpeth (NE613DZ). The soil was a sandy loam texture from the Rivington series. This is typical of many agricultural soils and supports a range of arable crops. A well was formed in the middle of the box and a cylindrical plastic container that matched the diameter of the sensor was used to support it at the level of the soil (Figure 2). This was done to prevent the moist soil from damaging the plate. The wired power supply and recording device were elevated on a shelf above the arena. The soil was kept visibly damp, but not wet, using distilled water. The arena was housed in an isolated laboratory at room temperature (15–21 °C). No other work was conducted in the laboratory during the recordings. Simultaneous video recordings were made using cameras (TLC 200 Pro, Brinno, Taipei City, Taiwan) and an infrared light; the camera was fixed to allow the entire arena to be seen in the viewfinder. The dampened pellets were glued to a microscope cover slip placed onto the centre of the sensor immediately prior to the slugs entering the trial.

Twenty slugs were placed on the soil; the location of slug placement was constant throughout all trials. Trials were set up around 16:00–17:00 and allowed to run into the next morning. Only hours 18:00 to 06:00 were analysed for continuity, as it was not possible to begin and end the trials at the same time, although this should be done if possible. Each trial was allowed to run regardless of pellet discovery or consumption. Immediately before or after each trial, a five-minute control recording was taken. Five trials were run for each of three formulations—non-toxic control, commercial metaldehyde, and commercial ferric phosphate—in a randomised block design. 

### 2.4. Audio Analysis 

Waveform Audio (WAV) files for each session were imported into Audacity 2.3.0—Audacity^®^. The control recording was used to create a noise profile for every session which allowed for background noise reduction. Individual trial WAV files containing the recording for one experiment were created. If feeding began before the defined beginning point for arena trials, these bites were disregarded. Individual trial recordings were imported into an application designed for analysing slug bites, which recorded the length and time of each bite.”

### 2.5. Statistical Methods

All statistical analysis was conducted using SPSS (26). 

#### 2.5.1. Laboratory Trials

To determine whether there was a difference between the mean length of bite between treatment groups and between individual slugs within treatment groups, a nested ANOVA was used. Outliers were measured by the inspection of a boxplot; if there were any present, they are described. Groups were checked for normality and homogeneity of variances using the Shapiro–Wilk (*p* > 0.05) and Levene’s Test (*p* > 0.05). Neither were reported unless the assumption was violated. Data are presented as mean ± standard deviation. For analyses that showed statistical significance, a Tukey HSD Test was used to identify significant differences between pairs of means. A Kruskal–Wallis test was conducted to determine whether there were differences in the number of bites between treatments groups. Distributions of bites were not similar, as assessed by visual inspection of a boxplot. Pairwise comparisons were performed using Dunn’s (1964) procedure with a Bonferroni correction for multiple comparisons. Adjusted *p*-values are presented.

#### 2.5.2. Arena Trials

A one-way Welsh ANOVA was conducted to determine whether there were differences in the number of bites between treatments groups. Distributions of bites were not similar, as assessed by the visual inspection of a boxplot. A Games–Howell post hoc analysis was used to determine pairwise comparisons. 

## 3. Results

### 3.1. Laboratory Trials 

An equal number of trials for each formulation was run, but the number of recordings where slugs fed (useable recordings) varied (Table 2). Recordings where no bites were recorded were not suitable for analysis. 

#### 3.1.1. Length of Bite

**Figure 3:**
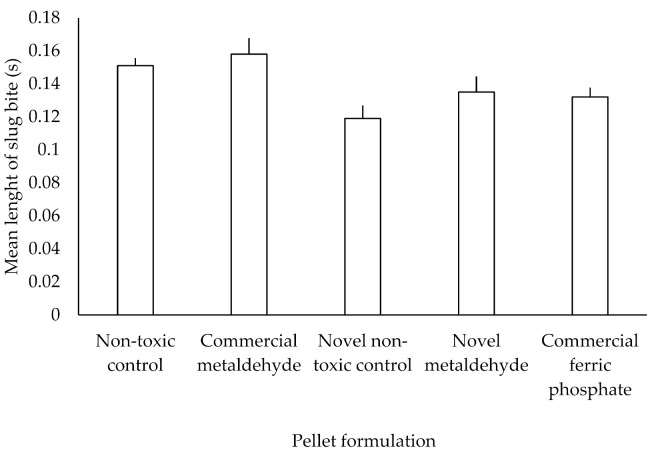
Mean lengths (+SD) of slug bites on different pellet formulations in laboratory trials. Non-toxic control (n = 24), commercial metaldehyde (n = 11), novel non-toxic control (n = 7), novel metaldehyde (n = 9) and commercial ferric phosphate (n = 10).

The mean lengths of slug bites between different formulations are shown in Figure 3. There were no significant differences in the length of slug bites between different formulations (F_4, 268_ = 1.012, *p* = 0.402), however, there was a difference between individual slugs within formulations (F_56, 731_ = 1.496, *p* < 0.05). 

#### 3.1.2. Number of Bites

**Figure 4:**
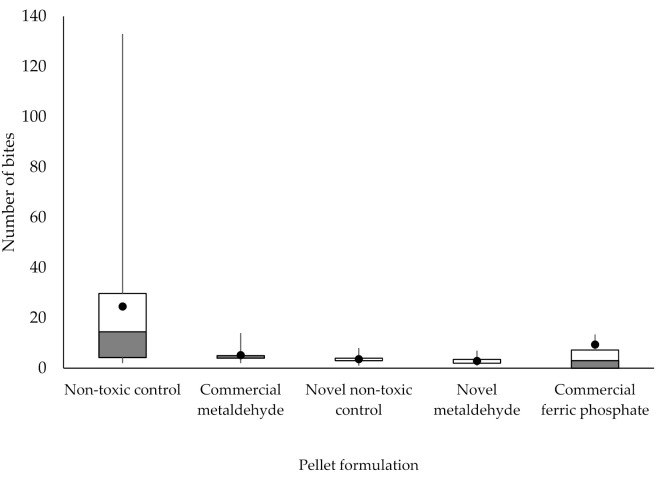
Mean number of slug bites on different pellet formulations in laboratory trials. Non-toxic control (n = 24), commercial metaldehyde (n = 11), novel non-toxic control (n = 7), novel metaldehyde (n = 9) and commercial ferric phosphate (n = 10). The box indicates the upper and lower quartiles, the whiskers indicate the range of data outside the upper and lower quartiles. The black circle indicates the mean.

The number of bites was not similar for all formulations, as assessed by visual inspection of a boxplot (Figure 4), and was significantly different, as χ2 (4) = 24.219, *p* < 0.001. The numbers of bites were significantly different between the non-toxic control and the novel non-toxic control pellets, the non-toxic control and the novel metaldehyde pellets, as well as the novel metaldehyde and commercial ferric phosphate pellets (*p* < 0.05). 

### 3.2. Arena Trials

Due to constraints on equipment access, it was not possible to run an equal number of recordings across the arena trials, non-toxic control (n = 5), commercial metaldehyde (n = 5) and commercial ferric phosphate (n = 4). However, all recordings were usable.

#### 3.2.1. Length of Bite

The arena trial was not conducted in a sound proofed environment, which resulted in a lower quality of recordings. Increased background noise or external sounds combined with the recording of the bites led to the recording of exceptionally high values (>0.4 s). This may also be due to the merging of several bites in quick succession or noise combining with the recording of the bite. The frequency of these outlying values was far greater in the arena trials than in the laboratory trials. Due to this, the length of bites for arena trials was not analysed. It WAs not anticipated that there would be a significant difference in the length of slug bites between pellet types in the arena trials. Disregarding all bites over >0.4 s, the average length of bites for the laboratory and arena trials was similar. We refer the reader to the discussion, for improvements that may be made to the methodology to improve recording quality in arena trials.

#### 3.2.2. Number of Bites 

**Figure 5:**
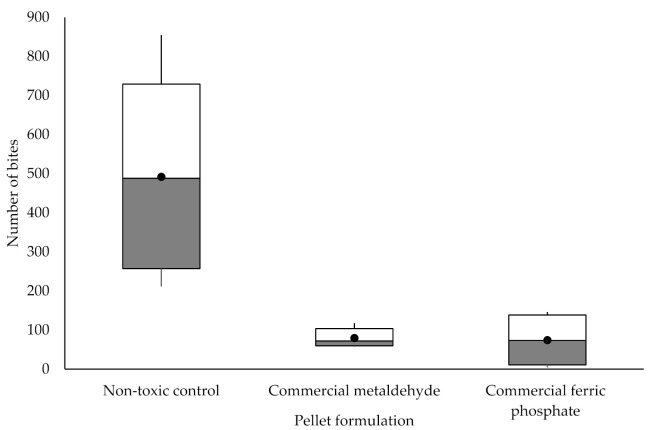
Mean number of slug bites on different pellet formulations in arena trials. Non-toxic control (n = 5), commercial metaldehyde (n = 5) and commercial ferric phosphate (n = 4). The box indicates the upper and lower quartiles, the whiskers indicate the range of data outside the upper and lower quartiles. The black circle indicates the mean.

The number of bites between pellet groups was statistically significantly different, as Welch’s F _(2, 5.118)_ = 5.790, *p* = 0.049. Statistically significant differences were observed between the non-toxic control and commercial metaldehyde as well as between the non-toxic control and commercial ferric phosphate (Figure 5).

## 4. Discussion

In our experiments, we found that *D. reticulatum*, when feeding on a range of pellets, did not show any change in the length of the bites between formulations, but did make fewer bites on pellets containing toxins or coated with a silica matrix. Slugs found non-toxic control pellets the most acceptable, resulting in significantly more usable recordings for this formulation. The lower acceptance of toxic pellets observed in laboratory trials differed with the literature where slugs were reported to nearly always accept the pellet regardless of molluscicide presence [34]. This could be due to different molluscicide formulations, general variation between the populations or experimental conditions. Additionally, previous experiments screened slugs to select those more likely to feed through the use of a dummy pellet [33,34,35,36]. A dummy pellet (any non-toxic pellet) is one that would be offered to a slug before entering the trial to test a willingness to feed, with only slugs willing to feed on the dummy pellet entering the trial. The use of a dummy pellet could mask the true results of acceptability between pellet types, by selecting slugs more likely to feed rather than representing a varied feeding spectrum, as would be found in field conditions. It was not disclosed how many slugs were excluded after refusing the dummy pellet to obtain the results, so we are unable to compare our results on acceptance with other work [33,34,35,36]. Our unpublished preliminary work found that the use of a dummy pellet prior to a trial was not useful for increasing the number of usable recordings for toxic or novel pellet types. It was observed that slugs that would accept a dummy pellet (non-toxic control) would reject a novel pellet or toxic pellet offered immediately after. 

The least accepted pellet was the novel non-toxic pellet, suggesting that slugs are deterred from feeding by the novel components in the formulation or from a by-product formed during the coating process. The main materials used to produce the novel coating were silica-based products (S. Newman, personal communication). Increased silica or silicon content has been observed to cause general health problems in animals, including molluscs [37,38,39,40,41]. In slugs, increased silicon content was suggested to cause reduced consumption [42,43]. This may be due to decreased leaf digestibility or wear on the feeding apparatus, with similar effects observed in other invertebrates [41,42,43]. However, the Golden Apple Snail, *Pomacea canaliculata*, showed no feeding aversion to plants with higher silicon content, indicating that an increased silicon content may not affect feeding in all mollusc species and that reduced feeding may be due to other factors [44]. Due to renewable teeth in molluscs, it could be argued that wear on the radula would only be a minor inhibition to feeding [44,45]. It is possible that the particle size of the silica in the novel products may be so fine that the product is not degraded further by the radula and that other components of the novel pellet may be the cause of aversion (S. Newman, personal communication). Further work comparing formulations with silica of different particle sizes could provide a better explanation of the results. 

Bite size was previously estimated from the body weight of the slug; slugs were allowed to take a set number of bites from a known quantity of food, before the meal was interrupted and the food reweighed [34,35]. When feeding on a non-toxic pellet, it was found that the bite size was not directly proportional to the weight and an allometric relationship was observed in *Deroceras* species [34,35]. Bite size also varied with the concentration of metaldehyde and between species [35]. Due to the difficulties in accurately measuring pellet and slug weight, we suggest that the size of the bite may be better reflected in the bite length (time where sound is produced during each bite). Longer lasting bites may reflect a higher food volume per bite, while shorter bites reflect a lower volume. While this has not been proposed before, there is merit in comparing the same characteristic (bite length) between formulations. One explanation for previously observed variation in bite size is a difference in consistency between pellets. Pellets of a softer consistency allow for greater volume in each individual bite, resulting in a greater total volume consumed in a fewer number of bites [35]. As all pellets in this experiment were more or less of the same consistency (subject to minor manufacturing variations), this may explain why no difference was observed between the length of bites between formulations. There was a difference between the length of bites between individual slugs within the same formulation. This could be related to the size of the slug which varied within formulations [34,35]

As the lengths of bites between pellet types were similar, we assume that variation in the amount consumed comes from the number of bites taken. A greater number of bites (assuming each at a similar volume) would suggest a larger (and usually longer) meal with more of the pellet consumed [35]. However, meal length was not considered a reliable indicator of consumption of pellets of different hardness, as softer pellets would be more readily consumed than harder pellets [34,35]. In our experiments, meal length was not considered as slugs were allowed in the laboratory trial for a set period. Further arena trials, with individual slugs feeding freely may present a greater understanding of meal length. Similarly, video recordings have been suggested to overestimate the length of the meal if the time in contact with the pellet was considered as the feeding time [35]. The consumption of pellets containing metaldehyde causes slug paralysis [26,35,36,46]. This could easily be misinterpreted by video methods as feeding, if the slug were to be paralysed in contact with the pellet. All slugs in the novel non-toxic control laboratory trials were observed to have made physical contact with the pellet, however, only 23% were recorded taking bites from the pellet. Ferric phosphate was not observed to cause immediate paralysis due to a different mode of action, which may result in more reliable video analysis.

The presence of a toxin (or novel component) decreased the palatability (number of bites taken) of a food substance, similar to the results found by other authors [33,34,35]. The paralysis of the feeding apparatus immediately after metaldehyde consumption may be the cause of low bite numbers in commercial and novel metaldehyde pellets [13,34,35]. No difference was observed in the number of bites taken between commercial metaldehyde, novel metaldehyde or the novel non-toxic control, suggesting that the novel pellets used in this study are not better at improving the palatability of pellets. No difference in the number of bites was observed between commercial metaldehyde and commercial ferric phosphate. This does not suggest that the products are equal when considering their usefulness as molluscicides due to different modes of action. Metaldehyde and ferric phosphate pellets are suggested to require distinct levels of consumption in order to be effective as molluscicides. Due to different modes of action, after a threshold amount is consumed, slugs were observed to take longer to die when consuming ferric phosphate when compared to metaldehyde. The observation of slugs that fed on any of the toxic pellets two to three days after the laboratory trials suggested that they did not consume enough pellet to cause mortality during the trials. Obtaining a greater number of recordings of toxic pellet types would improve the interpretation of these results. Further work could include longer feeding sessions to understand the relationship between the number of bites of a toxic pellet and paralysis as well as mortality. 

The main purpose of arena trials was to determine whether the artificial conditions in laboratory experiments had an impact on consumption. We were not able to collect any useable recording with novel pellet types. Preliminary work suggested that there may be difficulty collecting useful results due to lower chances of a slug encountering a single pellet while in the arena. This could be remedied with the use of multiple sensors with multiple food sources, increasing the likelihood of a slug encountering a pellet. Due to constraints on equipment and time, multiple slugs were used in each arena trial. Slugs took significantly more bites from non-toxic control pellets than on commercial pellets containing active ingredients—similar to the results observed in the laboratory trials. While it is not anticipated that the results would differ greatly, further work with individual slugs in an arena trial would allow the laboratory and arena experiments to be directly comparable. A further improvement of the arena trial methodology would be to conduct feeding experiments in a specialised recording room to improve sound quality and compare the results with the method presented in this paper or those taken in the field. It may also be possible that the feeding behaviour of slugs may have been altered by the temperature and humidity conditions within the laboratory [47,48,49]. Due to noise, it was not possible to conduct experiments in a controlled temperature room; the methodology could be improved with the use of a noise-reduced controlled temperature room. 

Within the limits of this study, no difference between toxic pellet types was observed. Poor acceptance and the consumption of the novel non-toxic control suggests that the components of the formulation deter feeding. Based on the results above, the reformulation of the novel pellet would be suggested and further work into the individual components of the novel formulation may be useful to the manufacturers. An ideal toxic pellet would show consumption similar to that of the non-toxic control. The methodology described has been useful in comparing pellet formulations and may be of use in the development of future pellet formulations.

## Figures and Tables

**Table 1:** Text reference, formulation name and active ingredient (AI) percentage (*w*/*w*) for pellets used in laboratory and area trials.

Text Reference	Formulation	AI Percentage (*w*/*w*)
Non-toxic control	Axcela*^®^* cereal	
Commercial metaldehyde	Axcela*^®^* metaldehyde	3
Novel non-toxic control	Silica coated Axcela cereal	
Novel metaldehyde	Silica coated Axcela metaldehyde	3
Commercial ferric phosphate	Sluxx HP*^®^*	2.97

**Table 2:** Number of useable and total bioacoustics recordings in laboratory trials.

Formulation	**Useable Recordings**	**Total Recordings**
Non-toxic control	24	30
Commercial metaldehyde	11	30
Novel non-toxic control	7	30
Novel metaldehyde	9	30
Commercial ferric phosphate	10	30

## Data Availability

The data represented in this study are available upon request from the corresponding author.
